# Wnt/β-Catenin signaling reduces *Bacillus Calmette-Guerin*-induced macrophage necrosis through a ROS -mediated PARP/AIF-dependent pathway

**DOI:** 10.1186/s12865-015-0080-5

**Published:** 2015-03-18

**Authors:** Xiaoling Wu, Guangcun Deng, Min Li, Yong Li, Chunyan Ma, Yujiong Wang, Xiaoming Liu

**Affiliations:** Key Laboratory of Ministry of Education for Conservation and Utilization of Special Biological Resources in the Western China, Ningxia University, 539 W Helanshan Road, Xixia District, Yinchuan, Ningxia 750021 China; College of Life Science, Ningxia University, 539 W Helanshan Road, Xixia District, Yinchuan, Ningxia 750021 China; Ningxia key laboratory of clinical and pathogenic microbiology, the General Hospital of Ningxia Medical University, 804 S. Shengli Street, Yinchuan, Ningxia 750004 China

**Keywords:** Wnt/β-catenin signaling, Alveolar macrophages, Cell death, Necrosis, Mycobacterial infection

## Abstract

**Background:**

Necrosis of alveolar macrophages following *Mycobacterium tuberculosis* infection has been demonstrated to play a vital role in the pathogenesis of tuberculosis. Our previous study demonstrated that Wnt/β-catenin signaling was able to promote mycobacteria-infected cell apoptosis by a caspase-dependent pathway. However, the functionality of this signaling in the necrosis of macrophage following mycobacterial infection remains largely unknown.

**Methods:**

Murine macrophage RAW264.7 cells were infected with *Bacillus Calmette-Guerin* (BCG) in the presence of Wnt/β-catenin signaling. The necrotic cell death was determined by cytometric assay and electronic microscopy; the productions of reactive oxygen species (ROS) and reduced glutathione (GSH) were measured by a cytometric analysis and an enzyme-linked immunosorbent assay, respectively; and the activity of poly (ADP-ribose) polymerase 1 (PARP-1)/apoptosis inhibition factor (AIF) signaling was examined by an immunoblotting assay.

**Results:**

The BCG can induce RAW264.7 macrophage cells necrosis in a dose- and time-dependent manner along with an accumulation of reactive oxygen species (ROS). Intriguingly, an enhancement of Wnt/β-catenin signaling shows an ability to reduce the mycobacteria-induced macrophage necrosis. Mechanistically, the activation of Wnt/β-catenin signaling is capable of inhibiting the necrotic cell death in BCG-infected RAW264.7 cells through a mechanism by which the Wnt signaling scavenges intracellular ROS accumulation and increases cellular GSH concentration. In addition, immunoblotting analysis further reveals that Wnt/β-catenin signaling is capable of inhibiting the ROS-mediated cell necrosis in part through a PARP-1/AIF- dependent pathway.

**Conclusions:**

An activation of Wnt/β-catenin signaling can inhibit BCG-induced macrophage necrosis by increasing the production of GSH and scavenging ROS in part through a mechanism of repression of PARP-1/AIF signaling pathway. This finding may thus provide an insight into the underlying mechanism of alveolar macrophage cell death in response to mycobacterial infection.

## Background

*Mycobacterium tuberculosis* (Mtb) is the cause of human tuberculosis (TB), which is regarded as one of the most harmful pathogens that is responsible for more deaths than any other microorganism. To date, one third of the population in the world has immunological evidence of Mtb infection [1]. TB is characterized by the presence of caseous necrotic lesions in the lungs, in which caseous necrotic lesions are mainly composed of cellular corpses that result from necrotic death in macrophages infected by Mtb [2]. Thus, necrotic death has been suggested to play a central role in the pathogenesis of TB, an inhibition of Mtb-infected cell necrosis is vital to the pathogenesis of TB disease. It has been demonstrated that the necrotic cell death, is associated with an energy independent and disordered cell death, which allows the release of viable mycobacteria for subsequent re-infection. Although several lines of recent studies suggested that necrosis could also follow a strictly programmed and ordered series of events [3,4], the precise mechanism underlying the necrosis of Mtb-infected host cells remains largely unknown.

A necrotic cell can be morphologically characterized by vacuolation of the cytoplasm, breakdown of the plasma membrane and an induction of inflammation around the dying cell attributable to the release of cellular contents and pro-inflammatory molecules. The necrosis of cells can be triggered mainly by cellular ‘accidents’ such as toxic insults, physical damage or reactive oxygen species (ROS) [5]. In this regard, ROS can act as an important mediator of cell death, and has strongly implicated in the aforementioned detrimental response by host that results in self-injury [6,7]. However, the molecular mechanisms underlying ROS-mediated cell death currently have not been fully demonstrated. There are several studies suggested that ROS was involved in the necrosis of many cell types [8,9]. For instances, Zhang et al. uncovered a role of receptor-interacting protein 3 (RIP3) in cell apoptosis/necrosis induced by tumor-necrosis factor (TNF)-α switching, by which cell necrosis could occur partly through an increasing energy metabolism–associated ROS production [10]. Such a ROS-mediated cell necrosis was also found in human hepatocellular carcinoma SK-Hep1 cells treated with β-lapachone, where β-lapachone could induce cell necrosis through an activation of ROS mediated RIP1 /poly ADP-ribose polymerase 1 (PARP-1)/apoptosis inhibition factor (AIF) signaling pathway [6]. However, recent studies demonstrated that the TNF-induced necrosis and PARP-1-mediated necrosis represented distinct routes to programmed necrotic cell death [11,12], suggesting a cell context-dependent and/or insult-dependent cell necrosis pathway.

The canonical (Wnt/β-catenin) pathway, have been evidenced to be involved in the interaction of Mtb and macrophage [13,14], and alveolar epithelial cells [15]. An increasing number of studies has demonstrated a regulatory role of Wnt signaling in cell apoptosis or cell death [16,17]. Our previous study also demonstrated that an activation of Wnt/β-catenin signaling was able to promote apoptosis of macrophage RAW264 cells infected with *Bacillus Calmette-Guerin* (BCG) [14]. However, the mechanism underpinning the modulatory role of Wnt/β-catenin signaling in cell death, in particular of necrosis of immune cells in response to various pathogen infections remains largely elusive. With this in mind, we thus interrogated the impact of the activation of Wnt/β-catenin signaling in the cell necrosis of macrophages in response to BCG infection using a murine macrophage RAW264.7 cell line.

## Methods

### Cell lines and Wnt3a conditioned medium

Murine macrophage RAW264.7 cell line was purchased from shanghai Institute of Biochemistry and Cell Biology (Shanghai, China); the Wnt3a producing cell line, L Wnt3a (overexpressing mouse Wnt3a, ATCC #CRL-2647) and its control L cell line (ATCC #ATCC #CRL-2648) were purchased from American Type Culture Collection (ATCC) (Masassas, VA, USA). The cells were cultured and maintained at 37°C in a humidified atmosphere of 5% CO_2_ and 95% air in DMEM medium (Invitrogen, Grand Island, NY, USA) supplemented with 10% Fetal Bovine Serum (FBS) and 1% pen/strep. The Wnt3a and control L cells were grown to confluence prior to be refreshed with DMEM/2% FBS and kept for 12 h. The culture media were collected and used for preparation of Wnt3a-conditioned medium (Wnt3a-CM) and control medium (control-CM), respectively. Since transformed cell lines were used *in vitro* in this study, informed consent was not required. There was not an ethnic concern either.

### Plasmids and transfection

The plasmid expressing Wnt inhibitor DKK1 pCS2-hDKK1-flag (Cat. #15494) was purchased from Addgene (www.addgene.com). For DNA transfection, RAW264.7 cells were seeded in 6 or 48 well plates and cultured for 18-24 h, and 80-90% confluent cells were used for transfection. The transfection was performed using FuGENE® HD transfection reagent per manufacturer’s instruction (Roche Biotechnology, Germany). The PARP-1 inhibitor (3-AB) (Sigma, St. Louis, MO, USA) was dissolved in Dimethyl sulfoxide (DMSO) at concentration of 20.0 mmol/L as stock solution. The final working concentration of 3-AB was 2.5 mmol/L. ROS scavenger N-acetyl-cysteine (NAC) (Sigma, St. Louis, MO, USA) was dissolved in water at concentration of 100 mmol/L as stock solution. The final working concentration of NAC was 10 mmol/L.

### Infection of RAW264.7 macrophage cells with BCG

*Mycobacterium bovis* BCG, Beijing strain (Center for Disease Control and Prevention (CCDC), Beijing, China) was grown at 37°C humidified incubator with shaking in Middle-brook 7H9 broth (BD Diagnostic Systems, Sparks, MD, USA) containing 10% albumin dextrose catalase supplement (Difco, West Molesey, Surrey, UK) for 2 weeks. Cultures were then harvested by centrifugation at 500 × g for 10 min and re-suspended in the medium. The bacilli were then titrated as previously described [18] prior to be aliquoted and stored at -80°C freezer. The above control or transfected RAW264.7 cells were infected with BCG at a multiplicity of infection (MOI) of 10 and incubated at 37°C in a 5% CO_2_, humidified air atmosphere for additional 6 h prior to be harvested for analysis.

### Flow cytometry analysis for cell necrosis

Cells were treated with different conditions for 2 h prior to be infected with BCG for additional 6 or 36 h before they were collected and stained with Annexin V and PI using an Apoptosis and Necrosis Detection Kit I (BD Pharmigen, San Jose, CA, USA) for flow cytometric analysis. The flow cytometry assay was performed on a BD FACSCanto II, and data was analyzed with FlowJo 8.8.6 software (Tree Star Inc, Ashland, OR, USA). All experiments were performed with biological triplicates and data are representative of at least three independent experiments.

### Electronic microscopy

The cells cultured under different conditions were first observed under an inverted microscope before being harvested for electronic microscopy analysis. For scanning electron microscopy (SEM) analysis, the cells were fixed with 2.5% glutaraldehyde, stained with 1.25% osmium tetroxide in PBS, dehydrated, and sputter coated prior to visualization on a Hitachi S-450 microscope (Tokyo, Japan); for transmission electron microscopy (TEM) analysis, the cells were fixed and stained as SEM, followed by infiltration with Spurr resin following dehydration. 80 nm serial sections were then viewed on a Hitachi H-7650 Electron Microscope (Tokyo, Japan). The apoptosis of cells was determined according the morphological criteria described in a previous study [19].

### Reduced glutathione (GSH) assay

RAW264.7 cells were treated with Control-CM, Wnt3a-CM, BCG, DKK1, LPS or H_2_O_2_ alone, or in combination. After 6 h incubation, the cellular reduced glutathione was quantified using GSH Assay kit (Jiancheng Institute of Biotechnology, Nanjing, China) per manufacturer’s protocol. The reduced GSH were normalized by protein concentrations. All experiments were performed with biological triplicates and data are representative of at least three independent experiments.

### Flow cytometric analysis of intracellular ROS

Cells were loaded with 5-(and-6)-chloromethyl-2-,7-dichlorofluorescin diacetate (DCHF-DA) for intracellular ROS measurement by accessing the intramitochondrial O_2_^-^ as described previously [20]. Briefly, the cells were harvested and washed with 1 × PBS, followed by incubation with 5 mmol/L DCHF-DA in dark for at 37°C for 15 min. The cells were then washed in 1 × PBS and resuspended in plain DMEM for flow cytometry assay. The flow cytometric analysis was performed on a BD FACSCanto II. At least 20,000 events were analyzed. All experiments were performed with biological triplicates and data are representative of at least three independent experiments.

### NAD^+^ analysis

5 × 10^4^ cells/well were seeded in a 96-well and culture overnight, before they are treated with different conditions. The total intracellular NAD^+^ was measured using the EnzyChrom NAD Assay Kit according to the protocol provided by manufacturer (E2ND-100, Bioassay Systems, Hayward, California).

### Immnoblotting analysis

Whole cell extract were prepared by homogenizing the cells in a lysis buffer (50 mM Tris-HCl, pH 7.5, 5 mM EDTA, 150 mM NaCl, 0.5% NP-40) for 60 min on ice. The lysates were then centrifuged at 10,000 × g for 10 min at 4°C, and the supernatants were collected as whole-cell extracts. The soluble protein concentration was measured with Bio-Rad Protein Assay (Bio-Rad Laboratories, Richmond, CA) using bovine serum albumin (BSA) as a standard. The cell extracts (50 μg) were separated by 10% sodium dodecyl sulfate (SDS)-polyacrylamide gel (SDS-PAGE) and transferred to a PVDF membrane (Millipore, Billerica, MA, USA). The membrane was blocked in 4% fat free dry milk in PBS containing 0.2% Tween-20 and probed using antibody against PARP-1, cleaved PARP-1 and AIF and β-actin followed by appropriate peroxidase labeled secondary antibodies. The blots were then developed using the enhanced chemiluminescence (ECL) reagent (Amersham Biosciences, Piscataway, NJ, USA). All above antibodies were from Cell Signaling Technology (Beverly, MA, USA).

### Statistical analysis

All data collected in this study was obtained from at least three independent experiments for each condition. SPSS18.0 analysis software was used for the statistic analysis. Statistical evaluation of the data was performed by one-way ANOVA and t-test for comparison of differences between the two groups. A value p < 0.05 set to represent a statistical difference and a value p < 0.01 set to represent a statistically significant difference. Data was presented as the mean ± standard deviations (SD).

## Results

### BCG induced-RAW264.7 cell necrosis can be inhibited by an activation of Wnt/β-catenin signaling

**Figure 1:**
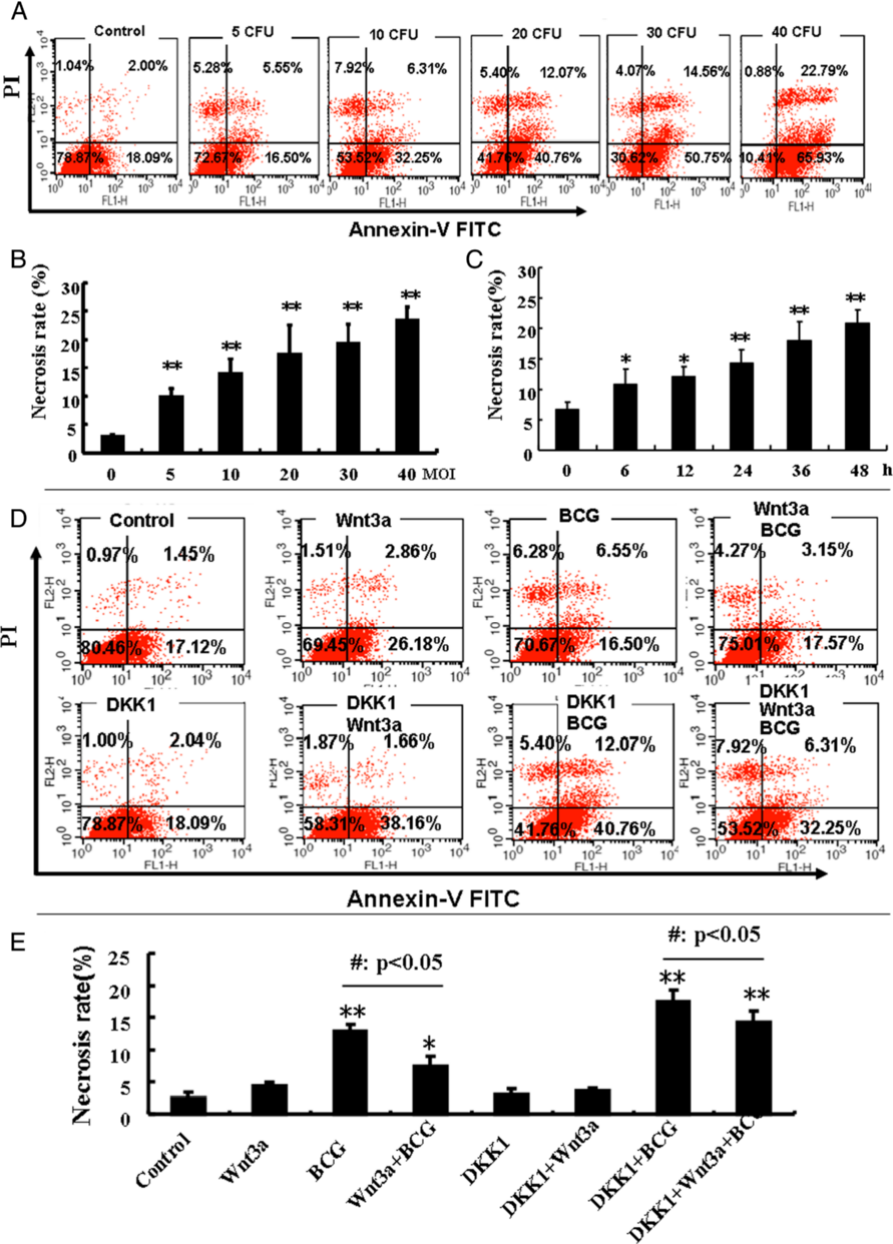
**The impact of Wnt/β-catenin signaling on necrosis of RAW264.7 cells in response to BCG infection. (A)** Representatives of scatter dot plot of flow cytometric analysis for the necrosis cell fraction for RAW264.7 cells treated with different doses of BCG. **(B)** A dose-dependent cell necrotic death of RAW264.7 cells infected with indicated dose of BCG for 6 h. The cell necrosis fraction was ascertained by a flow cytometry assay. **(C)** A time-dependent cell necrotic death of RAW264.7 cells infected with BCG. RAW264.7 cells were infected with BCG at MOI of 10 for indicated time. The cell necrosis fraction was ascertained by a flow cytometry assay. **(D)** Representatives of scatter dot plot of flow cytometric analysis for the necrosis cell fraction for RAW264.7 cells treated with indicated conditions for 6 h. The cells distributed in upper two quadrants were PI positive cells and considered as necrotic cell fraction. **(E)** Wnt/β-catenin signaling inhibits BCG-induced cell necrosis. RAW264.7 cells were infected with BCG at MOI of 10 or indicated conditions for 6 h prior to be used for flow cytometric analysis. Quantitative results revealed that an activation of Wnt/β-catenin signaling could inhibit cell necrosis, regardless of BCG infection. Compared to a Control-CM treated control cells, *: p < 0.05; **: p < 0.01. A control-CM treated compared to its corresponding Wnt3a-CM treated cells, #: p < 0.05; ##: p < 0.01, Data represented the mean ± SD from three independent triplicated experiments (N = 9).

**Figure 2:**
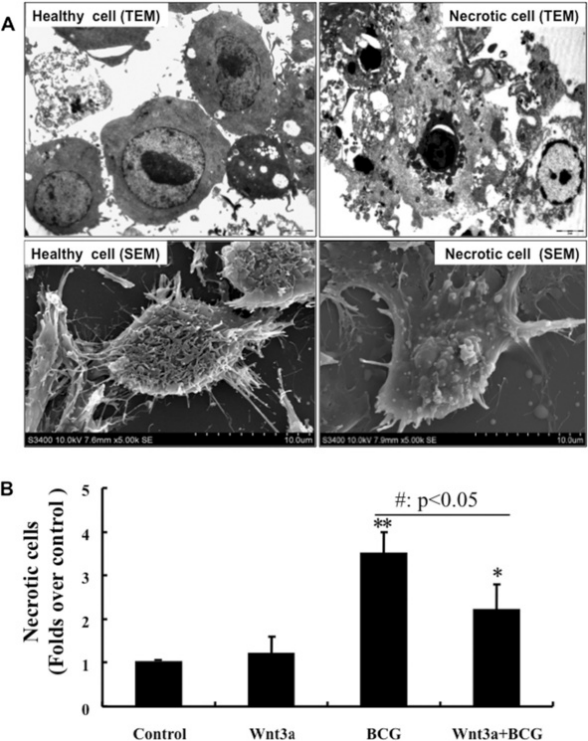
**Morphological analysis of the impact of Wnt3a on BCG-infected RAW264.7 cells necrosis.** RAW264.7 cells were exposed to Wnt3a-CM or control-CM, followed by infection of BCG at MOI of 10 for 24 h prior to be employed for EM analysis. **(A)** Representative images of TEM (top panel) and SEM (bottom panel) of healthy RAM264.7 cells (left panel) and necrotic cells (right panel). **(B)** Quantitative analysis of cells with a necrotic phenotype as determined by morphology using EM images. Control-CM treated cells compared to its corresponding Wnt3a-CM treated cells, #: p < 0.05; ##: p < 0.01. Data represented the mean ± SD from three independent triplicated experiments (N = 9). Bar in SEM images = 2 μm; Bar in TEM images = 10 μm.

In order to evaluate the necrosis of macrophages in response to mycobacterial infection, the murine alveolar RAW264.7 macrophage cells were infected with BCG at different dosages for varied time points. Results of flow cytometric analysis revealed a dose- and time- dependent reduction of cell viability and increased necrotic cell fraction following the BCG infection, indicating that BCG was able to induce RAW264.7 macrophage necrosis in a time- and dose-dependent manner (Figure 1A and B). Intriguingly, the BCG-induced cell necrosis could be significantly reduced when the cells were exposed to Wnt 3a, a ligand of the Wnt/β-catenin signaling (Figure 1C). On the contrary, cells enforced expression of Wnt signaling antagonist DKK1 exhibited an opposite effect of Wnt3a in RAW264.7 cells, where the introduction of DKK1 displayed a capacity to promote BCG induced-cell necrosis (Figure 1D). The function of Wnt/β-catenin signaling in inhibition of BCG-induced macrophage necrosis was further morphologically confirmed by accessing the characteristics necrotic cells using scanning electronic microscopy (SEM) and transmission electronic microscopy (TEM) (Figure 2A). The EM images of RAW264.7 cells revealed that the majority of cells exposed to control-CM showed healthy morphology, characterized with an integrity of nuclear membrane with abundant surrounding microvilli, uniform cytoplasm with rare cytoplasmic vacuoles, well-organized organelles, nuclei with clear membrane bounder, and uniform speckled distributed chromatin (Figure 2A and data not shown). While increasing numbers of necrotic cells was observed in cells exposed to BCG, which were characterized with loss of microvilli, disappearance of plasma membrane integrity and the presence of cellular organelles (Figure 2A and data not shown). Importantly, quantitative analysis demonstrated that the addition of Wnt3a-CM significantly could inhibit the BCG-infected cells to necrotic cell death, in comparison with the control-CM treated cells when the necrotic cell numbers were determined by an EM morphology (p < 0.01) (Figure 2B). These morphological results provided further evidence that activation of Wnt/β-catenin may inhibit necrosis in mycobacteria-infected macrophages.

### Wnt/β-catenin signaling suppresses the production of ROS in BCG-infected RAW264.7 cells

**Figure 3:**
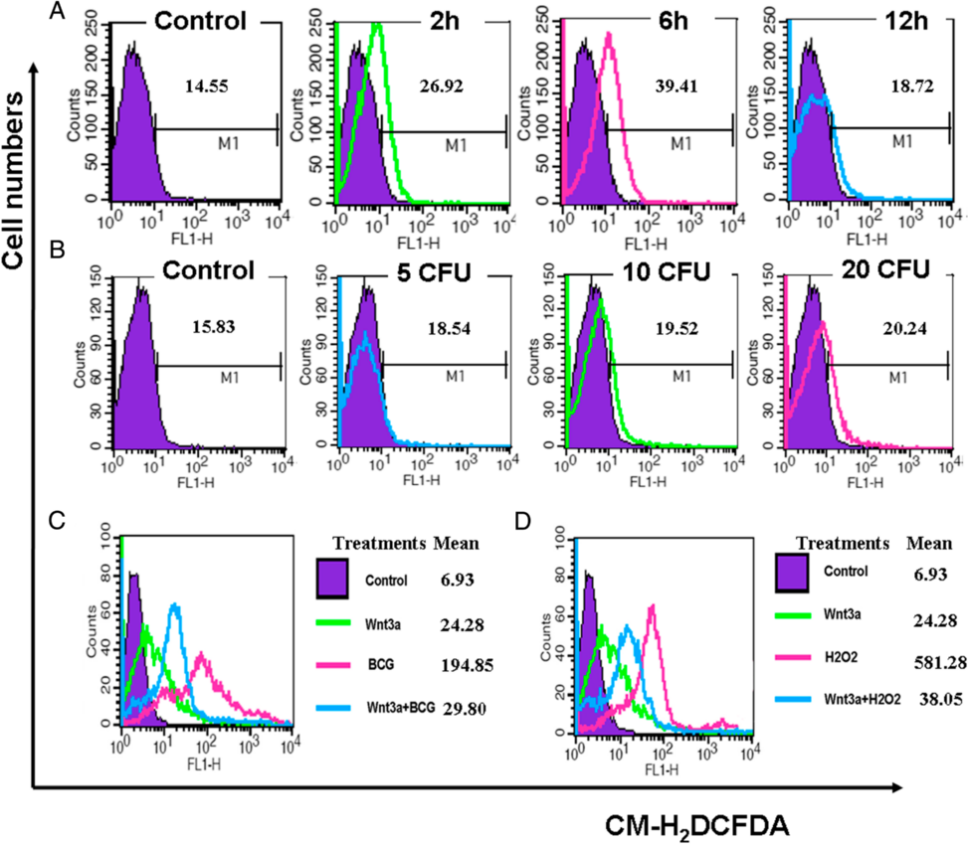
**Impacts of BCG and/or Wnt/β-catenin signaling on ROS production in RAW264.7 cells. (A)** RAW264.7 cells were infected with BCG at MOI of 10 for indicated time, then they were used for intracellular ROS measurement by a flow cytometry assay. A time-dependent ROS production was observed within 6 h. **(B)** RAW264.7 cells were infected with indicated doses of BCG for 6 h prior to be used for examination of intracellular ROS by a flow cytometry assay. A BCG dose-dependent ROS production was observed. **(C)** Impact of Wnt/β-catenin signaling on BCG-induced ROS production in RAW264.7 cells. RAW264.7 cells were infected with BCG at MOI of 10 for 6 h prior to be used for measuring intracellular ROS by a flow cytometry assay. An activation of Wnt/β-catenin signaling exhibited an ability to reduce BCG-induced ROS production. **(D)** Impact of Wnt/β-catenin signaling on oxidative stress in RAW264.7 cells. RAW264.7 cells were exposed to 500 μmol/L of H_2_O_2_ for 6 h before they were harvested for intracellular ROS measurement. The addition of Wnt3a showed a capacity to scavenge oxidative stressed-ROS accumulation.

**Figure 4:**
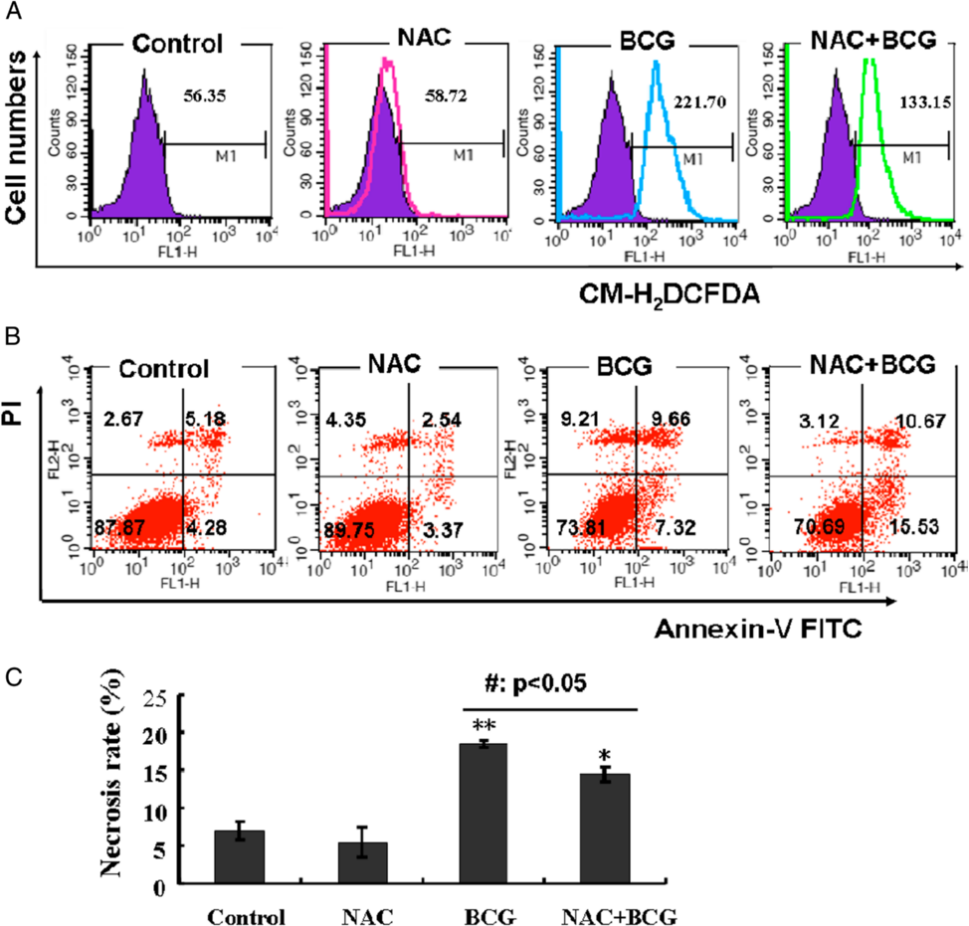
**ROS is involved in the BCG-induced necrosis in RAW264.7 cells.** The effect of ROS in the necrosis of BCG-infected cells was verified using a ROS scavenger NAC. **(A)** RAW264.7 cells were treated with BCG, NAC, or their combination, then they were used for intracellular ROS measurement by a flow cytometry assay within 6 h. **(B)** Representatives of scatter dot plot of flow cytometric analysis for the necrosis cell fraction for RAW264.7 cells infected with BCG, NAC, or their combination. **(C)** NAC inhibits BCG-induced cell necrosis. RAW264.7 cells were infected with BCG at MOI of 10 for 24 h prior to be used for flow cytometric analysis. Quantitative results revealed that NAC could inhibit cell necrosis induced by BCG. Compared to a control-CM treated cells, *: p < 0.05; **: p < 0.01; compared to its corresponding BCG treated cells, #: p < 0.05; ##: p < 0.01. Data represented the mean ± SD from three independent triplicated experiments (N = 9).

The importance of the balance between ROS production and scavenging is underscored by observations that oxidative stress can be either protective or damaging in several diseases [8]. With this in mind, we next examined whether the infection of BCG was able to induce RAW264.7 macrophages to produce ROS that subsequently induced cell necrosis. As expected, a dose- and time-dependent ROS production was determined in the RAW264.7 cells when they were infected with BCG at MOI of 20 or less for up to 6 h (Figure 3A and B). To verify whether BCG induces macrophages necrosis by ROS, a ROS scavenger NAC was employed to determine the impact of ROS on BCG-induced cell necrosis. As shown in Figure 4A, the addition of NAC (10 mmol/L) could significantly inhibit the BCG-induced ROS content, along with a decreased necrosis rate (p < 0.01) (Figure 4B,C). Of note, the increased level of ROS was correlated with the necrotic death of RAW264.7 cells in response to BCG infection.

Therefore, we next sought to explore whether an activation of Wnt/β-catenin signaling had an impact on the production of ROS. Noteworthy, a remarkable reduction of intracellular ROS production was observed in BCG-infected RAW264.7 cells that exposed to Wnt3a-CM (Figure 3C). The Wnt3a-mediated ROS scavenging activity was further confirmed by addition of H_2_O_2_ into RAW264.7 cell cultures at final concentration of 500 μmol/L, in which the Wnt3a-CM showed a capacity to dramatically reduce the ROS level (Figure 3D). These data imply that an activation of Wnt/β-catenin signaling can repress the BCG-induced cell necrosis through a mechanism of reducing the accumulation of intracellular ROS.

### Wnt/β-catenin signaling induces the production of Glutathione (GSH) in RAW264.7 cells

**Figure 5:**
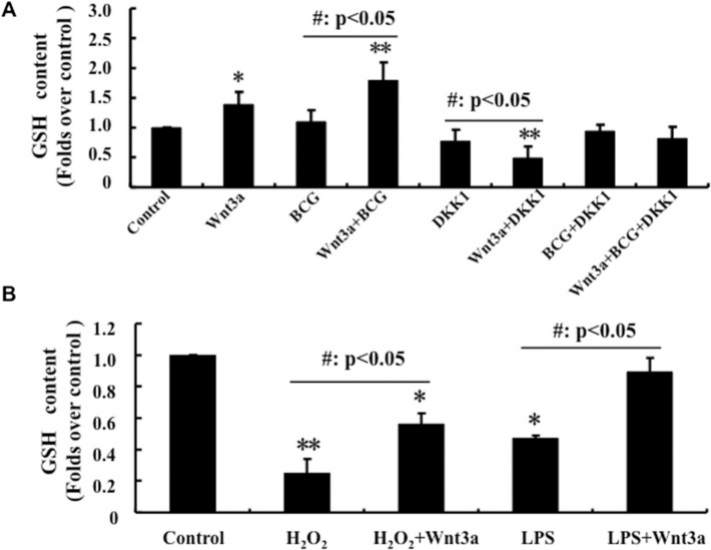
**Activation of Wnt/β-catenin signaling increases GSH concentration in RAW264.7 cells.** RAW264.7 cells were treated with BCG, Wnt3a, DKK1, H_2_O_2_ or their combination as indicated, they were then used for determination of intracellular GSH levels by an ELISA for GSH. **(A)** Impact of Wnt/β-catenin signaling on GSH production of RAW264.7 cells infected with BCG at MOI of 10 for 6 h. An activation of Wnt signaling by addition of Wnt3a-CM could induce GSH generation; in contrast, overexpression of Wnt inhibitor DKK1 reduced GSH levels. **(B)** Impact of Wnt/β-catenin signaling on GSH production of RAW264.7 cells exposed to oxidative stress H_2_O_2_ (500 μmol/L) or LPS (100 ng/mL) stimulation for 6 h. The Wnt signaling could increase intracellular reduced GSH concentration in cells stressed by H_2_O_2_ and LPS. Compared to a control-CM treated cells, *: p < 0.05, **: p < 0.01. Control-CM treated cells compared to its corresponding Wnt3a-CM treated cells, #: p < 0.05; ##: p < 0.01. Data represented the mean ± SD from three independent triplicate experiments (N = 9).

Next, we attempted to understand a possible underlying mechanism of Wnt/β-catenin signaling to scavenge the cellular ROS. Most recently, several studies demonstrated that the cellular ROS could be eliminated through detoxification mechanisms provided by endogenous antioxidant enzymes and antioxidants such as glutathione (GSH) [12,21]. This study reminded us to examine the GSH concentration in RAW264.7 cells treated with various conditions. The results exhibited that the presence of Wnt3a-CM could significantly increase the production of GSH in both of naïve RAW264.7 macrophages and the BCG-infected cells; in contrast, cells transfected with Wnt signaling antagonist DKK1 displayed an opposite function seen in the Wnt3a-CM treated cells, i.e. the expression of DKK1 could revise the Wnt3a-induced GSH productions (Figure 5A). To further confirm the capacity of Wnt3a to induce intracellular GSH production upon an external insults, the intracellular GSH levels of RAW264.7 cells treated with H_2_O_2_, lipopolysaccharide (LPS), H_2_O_2_/Wnt3a or LPS/Wnt3a were measured. Indeed, the addition of Wnt3a-CM could markedly increase the GSH levels in RAW264.7 cells treated with H_2_O_2_ or LPS (Figure 5B). These results clearly suggest that Wnt3a is able to dismiss intracellular ROS accumulation in RAW264.7 cells, which in turn may reduces the cell necrotic death induced by external stresses, such as BCG infection, oxidative stress of H_2_O_2_ or LPS stimulation.

### Down-regulated PARP-1 and AIF expression is involved in the Wnt/β-catenin signaling reduced BCG-induced necrosis in RAW264.7 cells

**Figure 6:**
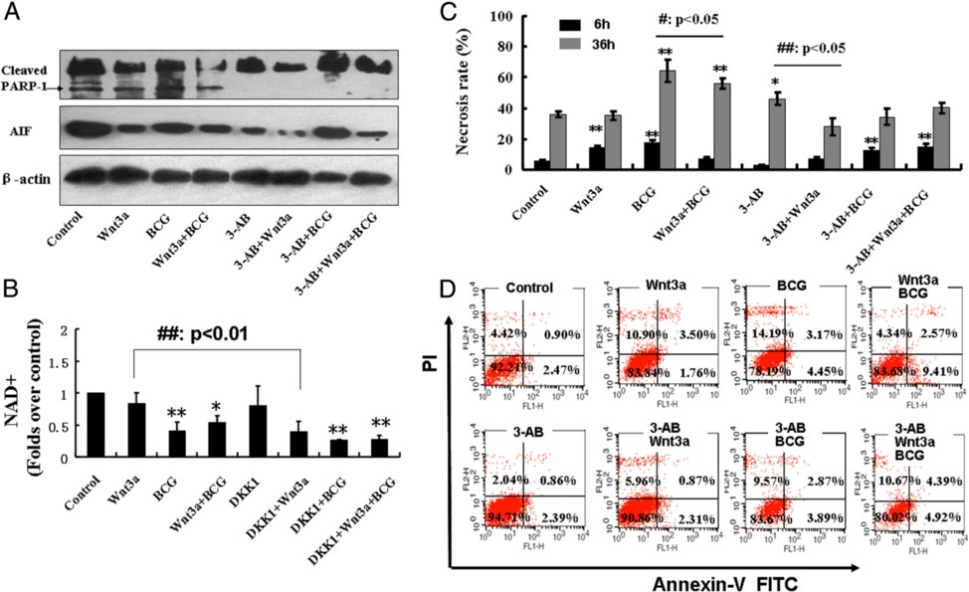
**PARP-1/AIF pathway is involved in BCG-induced RAW264.7 cell necrosis.** RAW264.7 cells were treated with indicated conditions for 24 h, the cells were then collected for immunobotting analysis or flow cytometric analysis. **(A)** Immunoblotting analysis for PARP-1 and its downstream mediator AIF proteins in RAW264.7 cells treated with indicated conditions for 24 h. Both Wnt3a-CM and PARP-1 inhibitor 3-AB displayed an ability to down-regulate PARP-1 and AIF. **(B)** NAD^+^ analysis for NAD^+^ in RAW264.7 cells treated with indicated conditions for 12 h. Wnt3a could inhibit the NAD^+^ depletion of RAW264.7 cells infected with BCG. **(C)** Flow cytometric analysis showed a time-dependent inhibition of BCG-induced necrosis mediated by Wnt3a or PARP-1 inhibitor 3-AB (2.5 mmol/L), suggesting the PARP-1 was involved in BCG-induced necrosis for RAW264.7 cells, and the Wnt signaling could inhibit RAW264.7 cell necrosis by down-regulation of PARP-1 activity. **(D)** Representatives of scatter dot plot from three independent experiments of flow cytometric analysis of the necrotic fraction of RAW264.7 cells treated with indicated conditions for 6 h. Compared to a control-CM treated cells * or #: p < 0.05, ** or ##: p < 0.01. Control-CM treated cells compared to its corresponding Wnt3a-CM treated cells, #: p < 0.05; ##: p < 0.01. Data represented the mean ± SD from three independent triplicate experiments (N = 9).

Since the PARP-1 has been demonstrated to contribute DNA base excision repair and maintenance of genomic stability [22]. A ROS-induced DNA damage could activate PARP-1; an over activated PARP-1 could rapidly utilize substrate NAD^+^ to transfer poly ADP-ribose (PAR) to itself and to nuclear acceptor proteins, subsequently the host cell consumes its ATP pools to resynthesize NAD^+^, which resulted in the host cell energy crisis and cell death [22,23]. Moreover, recently study suggested an involvement of PARP-1 in the ROS-induced cell necrotic death [6]. With this in mind, we thus interrogated the impact of Wnt/β-catenin signaling on PARP-1 of BCG-infected RAW264.7 cells by an immunoblotting analysis. The results showed an increased abundance of PARP-1 and its downstream signaling protein AIF, as well as the cleaved form PARP-1 protein in BCG-infected cells (Figure 6A). Of note, the addition of Wnt3a-CM could suppress the BCG-induced PARP-1 and AIF protein expression, and inhibit the PARP-1 activity by reducing the cleaved form of PARP-1 protein in RAW264.7 cells (Figure *6*A). Equal importantly, the pharmacological inhibitor of PARP-1, 3-AB also showed an activity to impair PARP-1 activation by inhibiting PARP-1 cleavage and AIF expression (Figure *6*A). To verify whether Wnt3a has an impact on the cellular NAD^+^ level, a marker of PARP-dependent cell necrosis, the cellular NAD^+^ content was detected. As shown in Figure *6*B, Wnt3a could inhibit the depletion of the BCG-induced cellular NAD+. On the contrary, an introduced expression of Wnt/β-catenin pathway inhibitor DKK1 could significantly reversed the effect of Wnt3a (Figure *6*B). In order to confirm whether the PARP-1 pathway was involved in the Wnt signaling-modulated necrosis in RAW 264.7 cells infected with BCG, the frequencies of necrotic death of RAW264.7 cells treated with BCG, Wnt3a, 3-AB alone or a combination were ascertained by a flow cytometric analysis (Figure *6*C and D). A time-dependent cell necrosis was found in RAW264.7 cells infected with BCG, more abundant necrotic cell death was observed at 36 h post BCG infection as compared with that at 6 h (Figure *6*C). Notably, the addition of Wnt3a-CM or PARP-1 inhibitor 3-AB alone could significantly reduce the BCG-induced cell necrosis (p < 0.01) (Figure *6*C and D. Interestingly, the addition of Wnt3a-CM into the 3-AB treated RAW264.7 cells failed to further inhibit BCG-induced necrosis (Figure *6*C and D). These results suggest that Wnt3a can inhibit necrosis of BCG-infected macrophage cells through the ROS-mediated PARP1/AIF signaling pathway.

## Discussion

A large body of study has demonstrated that Wnt signaling is capable of governing cell survival, proliferation, differentiation and apoptosis through multiple intracellular signaling pathways [24]. In this regard, the canonical Wnt signaling possesses a property of regulation of cell proliferation and apoptosis in a cell-context dependent manner, by which the activated Wnt/β-catenin signaling was able to enhance cell proliferation but also induce apoptosis in a variety of cells [16,17]. Our previous study also revealed that an activation of Wnt/β-catenin signaling could promote apoptosis for BCG-infected macrophage, in part through a caspase-dependent apoptosis pathway [14]. Along similar lines, in this study, we found that an activation of canonical Wnt signaling could inhibit the BCG-induced macrophage necrosis, at least in part through the ROS-mediated PARP1-AIF signaling pathway.

With regard to cell death induced by an external insult, oxidative stress and ROS are known to implicate in a number of physiological and pathological processes, leading to various biological consequences including necrosis [25]. In this context, free oxygen radicals are highly toxic to pathogens and are utilized as a tool for host cells to prevent colonization of tissues by microorganisms [7]. Therefore, ROS production has been recognized as one of the earliest innate immune responses of host cells in response to a microbial invasion. Despite the mode of ROS production may be cell context and insult-dependent, previous studies suggest that ROS can act as a common signal triggering cell death through the activation of MAPK and/or JNK pathway [9]. In agreement with these findings, in the present study, we found a dose- and time-dependent ROS production, along with an increased numbers of necrotic cell death in RAW264.7 cells in response to BCG infection. Equal importantly, an activation of canonical signaling by addition of Wnt3a-CM exhibited a decreased ROS production and cell necrosis in these cells, regardless of BCG infection. Such inhibitory role of Wnt/β-catenin in ROS production and necrosis was tightly correlated with suppressed expressions of PARP-1 and its downstream mediator AIF, and an inhibited PARP-1 activity. Such a PARP-1 signaling pathway induced necrosis was also found in human hepatocellular carcinoma SK-Hep1 cells [6] and HT-22 cells [26]. Most recently, Jiang et al. found that Wnt signaling pathway was involved in preventing steroid-induced osteonecrosis of the femoral head (steroid-induced ONFH) by suppressing PPARγ expression [27]. Conversely, a combination of Wnt signaling inhibitor DKK1 and hypoxia could cause a necrotic osteocytic cell death; and blocking of Dkk-1 was able to protect bone cells from glucocorticoid and hypoxia-induced cell injury [28]. Together with our findings, these studies suggest a cell context-dependent modulatory role of Wnt signaling in cell necrosis.

PARP-1 is a zinc finger protein that belongs to a family of 18 identified genes that transcribe poly (ADP-ribose) polymerases, enzymes that catalyze the covalent transfer of poly-ADP units from NAD^+^ to acceptor proteins. It is known as a key effector capable of amplifying necrotic signals in a necrotic pathway by which PARP-1 contributes to DNA base excision repair and the maintenance of genomic stability [22,29]. In addition, an increasing number of studies revealed that AIF was a mediator for cell necrosis. AIF could be translocated from the mitochondria to the cytosol and nucleus where it bound with DNA and RNA to induce caspase-independent chromatinolysis in the nucleus [30,31]. As GSH is an important intracellular scavenger for ROS, and the ratio between oxidized glutathione and reduced glutathione (GSSG/GSH ratio) has been used as an important index of the redox balance in the cell and consequently of cellular oxidative stress, an increased GSH level may be able to attenuate an oxidative stress [32]. Indeed, in the current study, an inverse correlation of GSH concentration and intracellular ROS level was observed in RAW264.7 cells treated with different conditions. Intriguingly, an activation of Wnt signaling could increase the concentration of intracellular GSH, subsequently reduced the ROS accumulation in cells infected with BCG. It is worthy to note that the infection of BCG did not lead a reduction of cellular GSH content in RAW264.7 cells, suggesting that the increased GSH content induced by Wnt3a might not the solo mechanism for elimination of BCG-induced ROS, despite the Wnt-induced intracellular GSH showed an ability to eliminate the BCG-induced ROS in this study. In addition, the process of ROS generation was complicated, for example, a BCG-up-regulated antimicrobial peptide cathelicidin LL-37could lead a rapid ROS production in human epithelial cells [33]. Moreover, NAD depletion is an essential event in the sequence leading from PARP-1 activation to cell death, in which NAD is required for maintaining PARP functions [34].

### Limitations

Certain limitations to our findings must be considered. Particularly an avirulent mycobacterial strain BCG and a murine macrophage cell line were used in this study. In future studies, primary macrophages and virulent mycobacteria should be employed to investigate the impacts of Wnt/β-catenin signaling in macrophages in response to mycobacterial infection and verify our current findings.

## Conclusions

The results reported in this study demonstrate that a ROS mediated PARP-1/AIF pathway is involved in the BCG-induced necrosis of alveolar macrophage RAW264.7 cells. Interestingly, an activation of Wnt/β-catenin signaling can inhibit the BCG-induced necrosis in part through the PARP-1/AIF pathway, by which Wnt/β-catenin signaling down-regulates expressions of PARP-1 and AIF, and induces the production of GSH that scavenges intracellular ROS accumulation. We thus uncovered a novel underlying mechanism of the Wnt/β-catenin signaling in necrotic death of macrophage in response to a mycrobacterial infection. Of note, other necrosis pathways may also be involved in the Wnt-mediated inhibition of cell necrosis, which need to be defined in future study. Further study on the regulation of Wnt/β-catenin signaling on macrophage necrosis induced by Mtb will be helpful for better understanding immune regulation and for developing new preventive and therapeutic strategies for TB.

## Abbreviations

Wnt: Wingless-type MMTV integration site family
Mtb: Mycobacterium tuberculosis
TB: Tuberculosis
BCG: Bacillus Calmette-Guerin
ROS: Reactive oxygen species
GSH: Reduced glutathione
PRAP-1: Poly (ADP-ribose) polymerase 1
AIF: Apoptosis Inhibition factor
3-AB: 3-aminobenzamide
